# A Laryngological Curiosity

**Published:** 1891-03

**Authors:** 


### A Laryngological Curiosity.

The *Medical Press*, December 3, 1890, states that at a recent meeting of laryngologists in London, Mr. Lennox Browne described the case of a middle-aged woman who was sent to him from the provinces for the purpose of deciding whether her malady was laryngeal cancer or phthisis. Though a tall, large-boned woman, she only weighed a little over ninety pounds, and was obviously very much emaciated. He peeped down her larynx and to his surprise saw what he recognized to be a plate with artificial teeth firmly impacted in the larynx, where it had been for the last twenty-two months unknown to the patient. She remembered having been awoke in the middle of the night by a violent fit of vomiting, and when the teeth were inquired after it was assumed that they had been thrown away with the dejections. From that day forth, however, she suffered from difficulty in breathing, pain on swallowing, etc., associated with progressive emaciation. With some difficulty the plate was removed and exhibited to the admiring friends, and the patient rapidly recovered health and spirits.

